# Towards a conceptual framework for addressing state-level barriers to decentralized clinical trials in the U.S.

**DOI:** 10.1017/cts.2023.584

**Published:** 2023-07-03

**Authors:** Stephanie J. Zawada, Kevin C. Ruff, Tara Sklar, Bart M. Demaerschalk

**Affiliations:** 1 Mayo Clinic College of Medicine and Science, Scottsdale, AZ, USA; 2 Mayo Clinic Center for Digital Health, Phoenix, AZ, USA; 3 University of Arizona James E. Rogers College of Law, Tucson, AZ, USA; 4 Arizona Telemedicine Program, Phoenix, AZ, USA

**Keywords:** Decentralized clinical trials, state policy, underserved populations, digital health

## Introduction

The challenge of adapting clinical trials to decentralized care models is increasingly pressing as virtual care becomes more common. In the United States, the patchwork of state and federal policies and regulations governing virtual care, such as limited interstate physician licensure, was a major barrier to the development of hybrid care models, blending in-person and telehealth, and the implementation of decentralized clinical trials (DCTs) [1]. During the COVID-19 Public Health Emergency (PHE), many states and the federal government enacted – often on a temporary basis – regulatory flexibilities to deliver care at home, including telehealth and remote monitoring, simultaneously empowering DCT models; however, the end of the PHE threatens to reverse leaps of progress achieved in the virtual care and DCT space during the pandemic [2,3]. Given the growing provider shortage and related decreased access to care, it is imperative that DCT researchers design trials with a state policy-conscious lens to prioritize diverse participant enrollment and overall retention [4–6]. Emerging work in this area demonstrates that engagement of patients, providers, and regulators in the design phase can identify barriers to DCTs [7–9]. Thus, strategies to facilitate interdisciplinary collaboration and the translation of stakeholder perspectives into actionable policy recommendations are needed.

Multiple existing frameworks are frequently employed to guide the process of identifying and addressing barriers to DCTs; however, none integrate perspectives from stakeholders across the translational spectrum [10]. As DCTs are a relatively new mode of conducting clinical trials, there is a paucity of literature examining their implementation. Moreover, the research available is preliminary, restricted by the first DCT recorded in 2011. While some DCT research evaluates pharmacological agents, others focus on remote monitoring [11–13]; regardless, DCTs are not limited by geographic restrictions, affording patients the option to participate from anywhere. Their scalable approach also has the potential to reduce patient burden, replacing in-person consults with telehealth, and improve the robustness of study data, capturing continuous *in situ* measurements using sensors [14]. No published research has specifically examined the role of state policies in the implementation of DCTs that enroll patients residing in-state.

We propose a novel conceptual framework to identify barriers to DCTs using stakeholder engagement that incorporates the broad perspectives of patients, their local providers, and state-based policymakers. Through the discovery of barriers experienced by these groups, this framework integrates the dynamic experiences of key stakeholders across the translational spectrum to identify and address policies that hinder the conduct of DCTs. Unlike previous frameworks, this framework addresses the hurdle of nonuniform policy landscapes that modulate the scope and scale of DCTs on a state-by-state basis in the United States. CARE-P [3]’s framework builds on previous DCT frameworks and is in the pilot implementation phase at Mayo Clinic.

## CARE-P^3^: A conceptual framework for identifying and addressing barriers to DCTs

**Figure 1.**
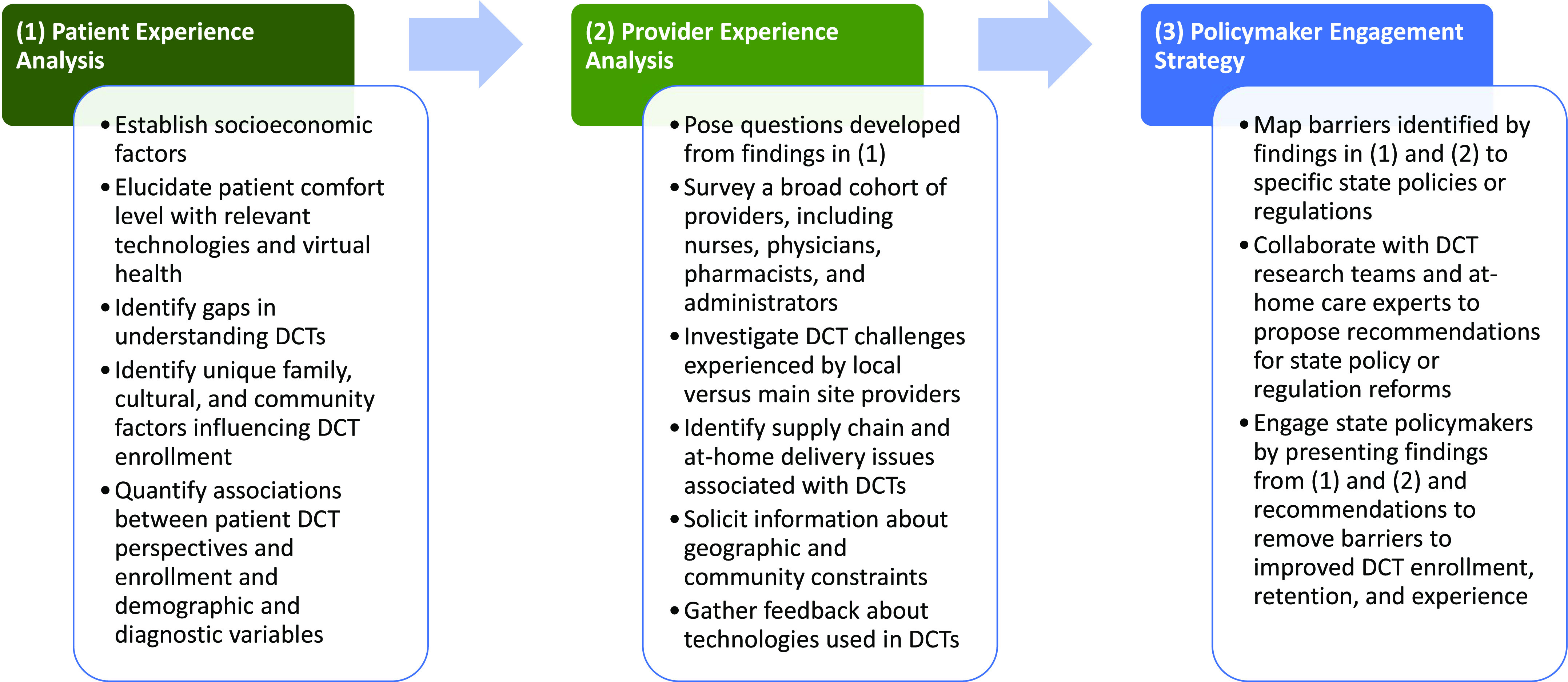
CARE-P^3^ framework.

Limited communication between scientists and policymakers has long been established as a barrier to integrating new technologies in clinical practice [15]. Additionally, it is critical to include the experiences of local communities, particularly those which are underserved, in identifying obstacles to virtual care [16]. The framework we describe below is the CARE-P^3^ framework, denoting: **C**linical **A**daptive **R**esearch **E**ngagement – **P**atients, **P**roviders, and **P**olicymakers. CARE-P^3^ uses stakeholder-engaged perspectives to identify barriers to DCTs and output actionable recommendations for state policymakers. This interdisciplinary framework engages patients and providers (Fig. 1), using mixed qualitative and quantitative methods to analyze their lived experiences, while considering the scope of reforms able to be implemented by state policymakers (Table 1).

**Table 1.** Stakeholders engaged and research needed to support the proposed framework

Stakeholder engaged (Phase)	Suggested research methods	Success markers
Patients eligible for DCT (1)	Qualitative semi-structured interviews	Community and sociodemographic influences are better understood
	Quantitative statistical analyses using baseline data and enrollment status	Relationships between DCT enrollment and patient admission status and demographic characteristics are elucidated
DCT Staff/Providers (2)	Qualitative semi-structured interviews using questions informed by findings from (1)	Research and practice concerns are better understood
		Staff/providers distinguish between patient conditions and technologies appropriate or inappropriate for DCTs
		Supply chain issues to deliver DCTs are better understood
Policymakers (3)	Apply action-research methods to map barriers identified in (1) and (2) to specific state policies/regulations	DCT researchers and expert staff have a better understanding of state-level barriers to DCTs
	Present findings from (1) and (2) with actionable recommendations from barrier-mapping to policymakers in open forum	State changemakers are provided with fit-for-purpose recommendations to facilitate improved DCTs
	Survey policymakers engaged about the relevancy of presentation and recommendations	Relevant policymakers have a better understanding of basic needs for DCT infrastructure

## Benefits of a stakeholder-engaged approach to barrier identification and remediation in DCTs

In the U.S., state laws frequently require healthcare entities and agencies to submit reports to the legislature [17]; yet, no publicly available reports examine the lived experiences of patients and providers that modulate their opinions and influence the adoption of DCTs [18]. Exploring barriers to DCTs beyond disciplinary silos, the CARE-P3 framework addresses a key knowledge problem faced by state policymakers programming the decentralized care ecosystem. For instance, the Federal Communications Commission, outlining strategies to expand access to remote care, advised state policymakers to remove barriers to internet services [19]; however, optimal policy reforms to increase access to the internet vary on a state-by-state basis, with underserved populations in some states also suffering from lack of electricity access [20]. Knowledge of the particular circumstances affecting DCTs in a given state at a point in time, such as sociodemographic barriers to internet access or restrictive prescribing policies linked with telehealth visits, must be gathered from dispersed groups in society, especially patients eligible for DCTs [21]. Including provider perspectives is critical, given their limited experience with emerging modes of decentralized care, a factor influencing their perceptions about patient eligibility for DCT recruitment [22]. Such a strategy provides robust evidence for state policymakers to identify and address barriers to DCT enrollment and retention.

## Conclusion

To implement, evaluate, and refine the CARE-P^3^ framework, DCT case studies characterized by a range of interventions and representative of various socioeconomic and diagnostic conditions are critical. While the pilot implementation of this framework has been trialed in both rural and urban settings, refining this approach will require multiple rounds of analysis and feedback to identify major gaps and arrive at a robust framework that can be used by researchers before, during, and after a DCT [22–24]. A chief challenge facing this work is limited research funding; however, evidence from the growing body of DCT research conducted during the pandemic may suggest that the economic impact of not addressing state-specific barriers will be more costly.

Depending on which temporary flexibilities introduced during the PHE are made permanent, this work may be more urgently needed for specific states or patient populations. Another challenge associated with this work is disparate terminology and communication styles that are stakeholder specific. For instance, language used by patients is not identical to terminology employed by providers. Similarly, policymakers use different terms than health care professionals and assess research findings with domain-specific methodologies [25].

Understanding the challenges faced by patients of different socioeconomic groups who elect or decline to participate in DCTs is crucial to diversifying clinical trials in the digital age. To develop safe and robust DCTs, it is essential that provider concerns and hurdles are addressed. Future research should also consider how to engage federal policymakers in the development of national DCT networks. Effectively communicating these findings and identifying actionable recommendations for policymakers are integral to developing equitable DCTs that yield high-quality data to fully realize the promise of decentralized clinical research.
